# Exploring User Needs for a Mobile Behavioral-Sensing Technology for Depression Management: Qualitative Study

**DOI:** 10.2196/10139

**Published:** 2018-07-17

**Authors:** Jingbo Meng, Syed Ali Hussain, David C Mohr, Mary Czerwinski, Mi Zhang

**Affiliations:** ^1^ Department of Communication Michigan State University East Lansing, MI United States; ^2^ Feinberg School of Medicine Northwestern University Chicago, IL United States; ^3^ Microsoft Research Redmond, WA United States; ^4^ Department of Electrical and Computer Engineering Michigan State University East Lansing, MI United States

**Keywords:** mobile sensing, mental health, depression, counseling, user-centered design

## Abstract

**Background:**

Today, college students are dealing with depression at some of the highest rates in decades. As the primary mental health service provider, university counseling centers are limited in their capacity and efficiency to provide mental health care due to time constraints and reliance on students’ self-reports. A mobile behavioral-sensing platform may serve as a solution to enhance the efficiency and accessibility of university counseling services.

**Objective:**

The main objectives of this study are to (1) understand the usefulness of a mobile sensing platform (ie, iSee) in improving counseling services and assisting students’ self-management of their depression conditions, and (2) explore what types of behavioral targets (ie, meaningful information extracted from raw sensor data) and feedback to deliver from both clinician and students’ perspectives.

**Methods:**

We conducted semistructured interviews with 9 clinicians and 12 students with depression recruited from a counseling center at a large Midwestern university. The interviews were 40-50 minutes long and were audio recorded and transcribed. The interview data were analyzed using thematic analysis with an inductive approach. Clinician and student interviews were analyzed separately for comparison. The process of extracting themes involved iterative coding, memo writing, theme revisits, and refinement.

**Results:**

From the clinician perspective, the mobile sensing platform helps to improve counseling service by providing objective evidence for clinicians and filling gaps in clinician-patient communication. Clinicians suggested providing students with their sensed behavioral targets organized around personalized goals. Clinicians also recommended delivering therapeutic feedback to students based on their sensed behavioral targets, including positive reinforcement, reflection reminders, and challenging negative thoughts. From the student perspective, the mobile sensing platform helps to ease continued self-tracking practices. Students expressed their need for integrated behavioral targets to understand correlations between behaviors and depression. They also pointed out that they would prefer to avoid seeing negative feedback.

**Conclusions:**

Although clinician and student participants shared views on the advantages of iSee in supporting university counseling, they had divergent opinions on the types of behavioral targets and feedback to be provided via iSee. This exploratory work gained initial insights into the design of a mobile sensing platform for depression management and informed a more conclusive research project for the future.

## Introduction

Today, college students are dealing with depression at some of the highest rates in decades. According to the 2017 National College Health Assessment, more than one-third of students had “felt so depressed that it was difficult to function” and more than two-thirds had “felt hopeless” within the previous school year [1]. Depression is associated with many other significant problems facing college students including alcohol and substance abuse, eating disorders, dropout, self-injury, and suicide [2]. Responding to this mental health issue is imperative on college campuses.

University counseling centers are the primary source for students to access mental health care on college campuses. Unfortunately, there is a lack of capacity and efficiency for university counseling centers to provide counseling services to all students whenever they need. With regard to capacity, the demand of students with depression concerns have been rapidly increasing [3], outpacing the number of staff and working hours that counseling centers could provide. For efficiency, clinicians’ reliance on students’ self-reports may delay accurate depression assessment and effective treatment delivery. Students with depression are likely to miss clinical appointments [4] or are unable to provide clear or complete information for a variety of reasons, for example forgetfulness or embarrassment [5]. When patients’ self-reports are unavailable, or not able to provide an accurate and complete profile about the patients, clinicians may have a difficult time assessing patients’ depression conditions, monitoring therapy outcomes [6], and delivering effective therapies.

**Figure 1:**
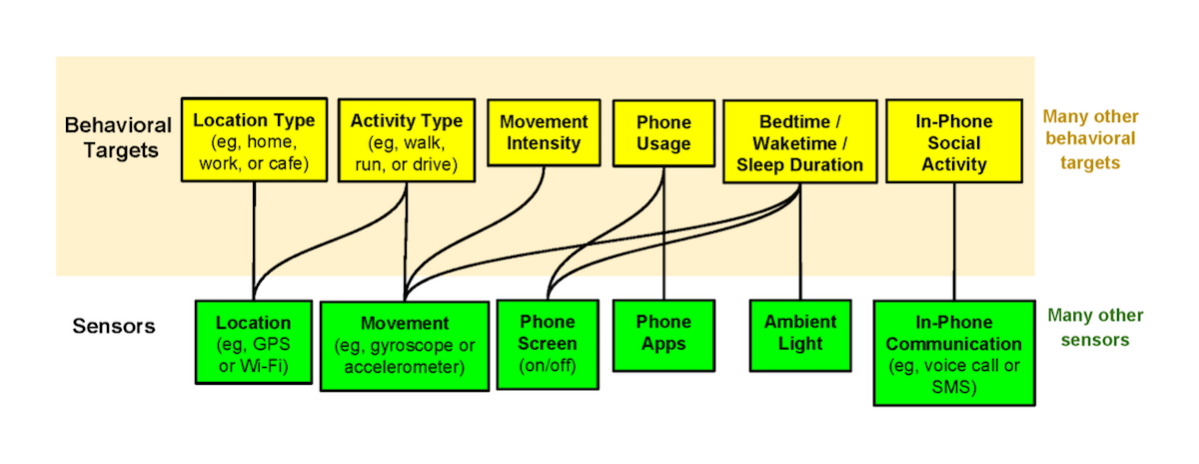
An example of sensemaking behavioral targets [7]. GPS: global positioning system; SMS: short message service.

We propose a large project that aims to design, develop, and implement a mobile sensing platform to address the current challenges that university counseling centers are facing. As the first step of the large project, the present study focuses on gaining insights into the usefulness and design of a mobile sensing platform in enhancing the counseling services available. A mobile sensing platform consists of three components. First, the platform relies on a variety of sensors on mobile and wearable devices which detect and measure physical properties of humans and their environment [7]. For example, smartwatches and smartphones contain onboard sensors that track people’s location, movement, sleep, and communication, as well as light and sound in the environment. Second, the platform converts the raw sensor data into behavioral targets through data analytics algorithms [7]. Behavioral targets are meaningful constructs measured by the raw sensor data. For example, raw sensor data may detect ambient light, sound, body movement, and whether the phone screen is on or off and potential behavioral targets can be extracted with regard to bedtime or waketime and sleep duration. These behavioral targets can serve as indicators of depressive symptoms (see Figure 1). Third, the platform delivers behavioral targets and feedback to clinicians and student users.

Existing research has documented the benefits of using mobile sensing technology for mental health research [7-10]. Among the pioneer projects, the MONARCA project used a variety of phone sensors, such as GPS (global positioning system) and an accelerometer, to detect the mental states of bipolar patients [11]. The StudentLife project used mobile sensing technology to monitor the daily behavior of college students and found that the tracked behaviors were associated with students’ mental states, such as stress [12]. The CrossCheck project used data collected from phone sensors and ecological momentary assessment to build models to predict mental health indicators in schizophrenic patients based on phone sensor data [8]. In addition, mobile technologies have been shown to have advantages in delivering mental health therapies [13,14]. Systematic reviews have summarized the therapeutic effects of technology mediated mental health information systems [15]. For example, mental health apps on mobile phones could improve the accessibility to treatment and facilitate proactive seeking for professional help [13,16].

Building on previous research, this study represents the first effort toward applying a mobile sensing platform in enhancing mental health services at university counseling centers. The purpose of this study is to explore and gain initial insights into the usefulness and design of the mobile sensing platform using a qualitative approach. Specifically, the first purpose of this study is to understand the usefulness of a mobile sensing platform in improving counseling services and assisting students’ self-management of their depression conditions.

Moreover, a variety of behavioral targets could be extracted statistically using algorithms and machine learning [7]. However, domain expertise and human intelligence are crucial for constructing meaningful behavioral targets. Therefore, the second purpose of this study is to explore what types of behavioral targets and feedback are helpful from both the clinicians’ and students’ perspectives.

## Methods

### Participants

We recruited clinicians and student participants from the counseling center at Michigan State University (MSU). Recruitment occurred between February 15, 2017 and March 30, 2017. To recruit clinician participants, an invitation email with information about the study was sent to all 21 clinicians by the director of the counseling center and 9 clinicians responded to sign up for the study. To recruit student participants, a flyer was posted on the wall of the waiting room at the counseling center. To be an eligible participant in this study, students needed to meet the following criteria: (1) be 18 years old or older; (2) currently enrolled as a college student; (3) having been diagnosed with moderate, moderate-to-severe, or severe depression; and (4) are currently receiving college counseling services for their depression condition. Twelve eligible student participants signed up for the study. Each clinician and student participant received US $15 as compensation for participating the study. All participants had to sign a consent form in accordance with a study protocol approved by the MSU Institutional Review Board.

For the 9 clinicians (aged 31-55 years; mean 42 years, SD 5.83; 8 female), 3 were clinical psychologists, 3 were clinical counselors, 2 were educational psychologists, and 1 was a clinical social worker. For the 12 student participants (aged 19-22 years; mean 21, SD 1.22; 7 female), 5 were mildly depressed with PHQ-9 (PHQ-9 is a diagnostic assessment of major depression disorder) scores ranging from 9 to 14, and 7 were moderately depressed with PHQ-9 scores ranging from 15-19. Out of the 12 student participants, 10 were undergoing treatment (ie, antidepressants) with the student health center and all of them were receiving psychological counseling at the MSU counseling center or counseling service outside the MSU campus.

### Procedures

**Figure 2:**
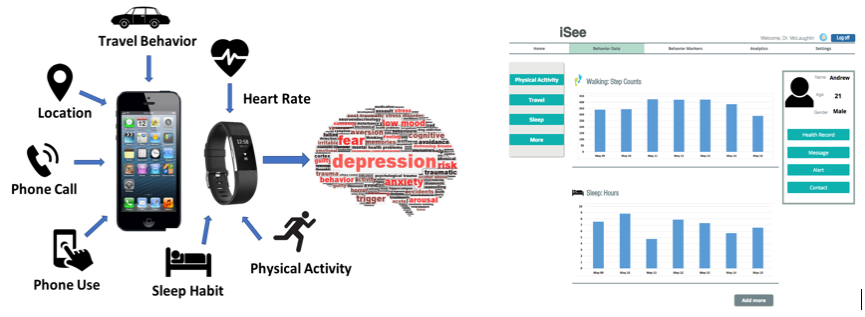
A conceptual prototype of iSee sensing platform and interface for clinicians.

**Figure 3:**
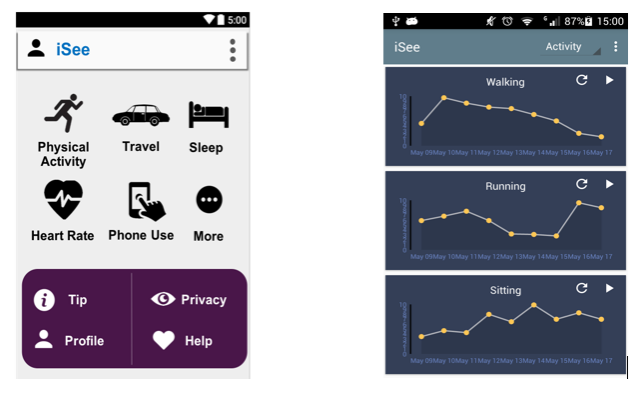
A conceptual prototype of iSee interface for students.

We conducted semistructured interviews with all of the participants and the interviews lasted between 40 and 50 minutes. Interviews with clinicians were conducted in their offices; interviews with student participants were conducted at a location of their choice. The interviewees were informed that the background of the study was to develop and design a mobile sensing platform named iSee. The interviewees were informed that the iSee system leveraged the mobile sensing capacity of the mobile phone to collect raw sensor data related to physical properties of humans and environment. The iSee system then used data analytics to convert raw sensor data into meaningful behavioral targets, and then deliver behavioral targets information through a dashboard to clinicians and through a mobile app to students. For the interviews with the clinicians, the interviewer first described iSee and then displayed an example of a representation of sensed data from a student’s phone that might be presented on a clinician’s dashboard (Figure 2). For the interviews with the student participants, the interviewer first administrated PHQ-9 with paper and pencil to characterize the sample, then described iSee, and finally presented an example of output of sensed data on a mobile app (Figure 3). The interviewer explained to clinicians and students that the figures presented were only conceptual prototypes of iSee, and the purpose of the interview was to solicit their thoughts and opinion about the usefulness of iSee, what sensed behavioral targets they preferred, and how to present them via iSee.

After the introduction, three general lines of inquiry were pursued using semistructured interviews: (1) how the mobile sensing platform might be useful to counseling service (clinicians) and depression management (students), (2) what behavioral targets should be provided and in what format they should be provided to maximize the usefulness, and (3) identification of barriers to using the mobile sensing platform for clinicians and students (see interview protocols in Multimedia Appendix 1). All the interviews were audio recorded.

### Data Analysis

All interviews were transcribed, and the interview data was analyzed using thematic analysis with an inductive approach [17]. Three researchers read the transcripts, familiarized themselves with the data, and independently generated an initial list of codes which represented the most basic elements in the raw data. In the next step, the same 3 researchers met frequently to discuss their initial codes, group initial codes into larger categories, and extracted the underlying concepts in the initial codes. In the subsequent analysis, they searched for themes, sorted the different categories and concepts into potential themes, and collated all the relevant coded data extracts within the identified themes using Nvivo 11. The last step of the analysis focused on reviewing all the collated extracts for each theme, considering whether they appeared to form a coherent pattern, and examining the validity of individual themes in relation to the entire dataset. This process involved iterative coding, memo writing, and constant comparison of the data to the emerging themes. The three researchers have background in health communication, which may affect their data interpretation and choices of themes.

## Results

### Clinician Perspective

We first report the findings from the clinician perspective with respect to the usefulness of iSee in improving college counseling service for depression management, the preferred behavioral target data and feedback, and concerns about using iSee in counseling practice.

#### Usefulness of iSee in Counseling

##### Objective Feedback to Students with Depression

Seven clinicians believed that behavioral targets tracked via the mobile sensing platform would provide students with an objective check against their subjective beliefs about their behaviors.

...for people with depression, the subjective life experience is often different than the objective. You may think you’re not eating enough, but you’re eating too much; or you might think you’ve walked enough, but really you haven’t, and instead, you’ve lain on bed for 14 hours.

Clinician 1

In particular, clinicians explained that depression could create a tendency to view things more negatively.

My clients always think negatively about themselves because that is what depression does. So, having the tracked data might help them do reality check. Like, you say have no friends and nobody likes you, but you have these many text messages exchanged with your friends.

Clinician 4

##### Objective Evidence to Clinicians

Seven clinicians said that they would assign homework (eg, increase physical activity) for students to complete between counseling sessions. Clinicians spoke about how the mobile behavioral-sensing platform could help them confirm students’ compliance and adherence to the assigned homework.

I’m big on physical activity when treating depression...the data can help me to see if they are complying with the 45 minutes four days a week, then I can say confidently that we have done a serious intervention that would like parallel what would happen with a medication like an SSRI [selective serotonin reuptake inhibitor].

Clinician 1

Moreover, the mobile sensing platform can be useful to assist clinicians’ decision-making about what homework to assign to students.

I’d like to see my clients’ progress in completing the homework. Automatic tracking is helpful because I can go check and adjust the goals for them. Working towards small and achievable goals is very important.

Clinician 9

It’s important to set an appropriate homework for them because if they cannot complete, they will see themselves fail, and that feeds their depression.

Clinician 3

##### Filling Communication Gaps

From the interviews, we learned that a counseling session typically ran for approximately 45-50 minutes. Due to the time limit, there may not be sufficient communication between clinicians and students. Five clinicians mentioned that, during one counseling session, they tended to focus on topics such as teaching mindfulness or dealing with a mental breakdown, leaving little time to communicate with students about their depressive symptoms such as sleep disruption and social avoidance. These clinicians said that the behavioral targets provided by the mobile sensing platform could complement clinician-student communication during the counseling session.

We have a progress note for each client, like asking about their sleep and eating when we start or end a counseling session. But sometimes we don’t or forget to check progress because we are focusing on certain things like crises. But if it’s a crisis, we really have to know the basic ones. The data can be a good reminder and it actually saves a lot of time for us.

Clinician 3

#### How to Provide Behavioral Targets

##### Data Provision to Students under Clinician Guidance

Three clinicians expressed a strong hope to have the mobile sensing platform developed and used under their guidance. This may help to maximize the benefits of showing data to students with depression while minimizing the potential risks.

If clients have never received counseling service before, and they might not have tolerance to reflect their own behaviors, telling them that their sleep quality is poor could be depressing. I would take a reserved stance in terms of how much and the kind of data given to clients, and when, depending on their conditions.

Clinician 6

##### Goal-Oriented Presentation of Behavioral Targets

Four clinicians suggested organizing behavioral targets around personalized goals. Clinicians would like to work with their clients to set up small and achievable goals (eg. go to bed at 11 pm and get up at 7 am) to manage sleep dysregulation in depression. Behavioral targets can be shown in terms of students’ baseline and on-going progress towards their goals.

The best way to provide data to a client is to give them their baseline data, and then have them put in their goals, and then give them a readout at some regular interval of are you meeting your goal or exceed your goal.

Clinician 1

Clinicians can help to put an appropriate goal for a client. Instead of giving some scores out of context, it will be helpful to tell clients how they are doing compared to their baselines.

Clinician 7

#### How to Provide Feedback

Five clinicians pointed out that the value of the mobile sensing platform is that it can provide one’s real-time behavior and constant behavioral targets, which serves as the basis of personalized feedback. A weekly counseling session takes up, at most, one hour, leaving 167 more hours per week where students with depression are on their own. Feedback based on students’ tracked behavior could be delivered outside the counseling sessions by clinicians or the iSee platform. These clinicians have mentioned that iSee could serve as an extension of counseling service beyond working hours.

##### Positive Reinforcement

The first type of feedback which emerged from our interview data is positive reinforcement in the form of esteem support messages, pointed out by 5 out of 9 clinicians. Esteem support messages defined as are words that acknowledge and validate one’s self-worth and achievement [18]. When students are following clinicians’ advice and making good progress in managing their depression conditions, positive reinforcement can boost their self-esteem and motivate them to continue.

I would like a portal for me to be able to go in and give them notes of encouragement. Like “Hey, John, saw your data today. You look great.”

Clinician 1

Or if clinicians do not have time to review the behavioral target data, the system might automate message delivery based on the student’s tracked behavior. For example, Clinician 8 suggested using the iSee platform to send the support message:

When clients are doing well, they may get an alert that says good job or congratulations. They will feel good about themselves.

Clinician 8

##### Reflection Reminder

Instead of showing students behavioral targets that might be demoralizing, 3 clinicians suggested that gentle reminders may be used to raise attention to a condition and encourage reflection.

When the tracked data do not look very good, you could use popup reminders that they can ask themselves questions. Like, “did you do things you need to do to take care of yourself today?” or “do you know that when your sleep is not well, you need to watch out because you might feel down this week?”

Clinician 4

These reminders may provide reassurance, activate students’ reflection, or encourage preventive actions.

##### Challenging Negative Thoughts

Negative thoughts are a hallmark of depression. Clinician 9 pointed out that to review one’s behavior trend could provide a context to challenge one’s negative perception:

When students are depressed, they feel critical of themselves. Instead of saying, “Yay, it’s great! I moved three times today.” They are like, “I only moved three times today. This is the worst thing I’m feeling.” So, at this point of time, we can provide alternative and more positive thinking.

Clinician 9

#### Barriers: Time and Liability

Time and liability are the two major barriers of using the mobile sensing platform at the counseling center. Two clinicians mentioned that they had very busy schedules, so they were not sure whether they would have time to review the data consistently. In addition, 3 clinicians were not entirely clear about their responsibilities to patients after having their behavioral targets information.

...it can be bad because if we missed something, or we can’t reach them, then we are still responsible for the information that we have. It can go both ways, but it’s just important to think about the liability piece.

Clinician 3

### Student Perspective

In this section, we report the findings from the student perspective with respect to the usefulness of iSee in managing depression, the preferred behavioral targets and feedback, and concerns about using iSee in everyday life.

#### Usefulness: Support Continued Self-tracking

Nine out of the 12 student participants interviewed mentioned that they had used some kind of mobile tracking apps but unfortunately, it was difficult to continue the self-tracking practice. The discontinuance of self-tracking is mainly due to the amount of effort and time spent on inputting one’s data.

I’ve tried tracking myself, but I stopped. The hardest part was logging the diet and exercise because there are so many variables that go into it.

Student 4

I used some trackers, but you had to input all of that data. I used that for a while but stopped because that’s just like another thing to do.

Student 7

In particular, for students with depression, they may lack the motivation and energy to do self-tracking.

When I am going through a cycle of depression, I’m like, “oh, I’m so depressed now. Tracking is going to go by the wayside. It’s just like not something on top of my list.”

Student 7

#### Need for Data on Integrated Behavioral Targets

Seven out of the 12 student participants pointed out that they found data that could explain the correlations between various behavioral targets and their depression would be helpful to increase self-awareness.

I find nights that I noticeably don’t sleep well, I have a worse mood when I wake up, so sleep tracking is important to me. I want to know how much I wake up versus how long I sleep, and how that affects me.

Student 1

I find running on treadmill and lifting weights is a way to relieve stress. It will be great if I can keep track of that and notice, hey, I feel happier today because I worked out.

Student 10

...it’s good to kind of mentally be aware like, “wow, I ate this much food.” I tend to eat more when I’m more depressed. It’s more of a comfort food. Visualization will be helpful so that I can see really highs or lows.

Student 11

#### Avoid Negative Feedback, But Seek Positive Feedback

Four student participants tried to avoid seeing negative feedback because of the increased feeling of helplessness.

I feel like just get discouraged when you always are giving me the same results, or the same thing is happening. I want some type of improvement, but if isn’t any, I don’t want to know.

Student 12

...isn’t depressing to see my condition get worse? I would dismiss the data if I knew it would tell me something bad.

Student 8

In contrast, 6 student participants expressed their love and need for positive supportive messages.

...some type of motivation or support and even if you had struggles, it wouldn’t say “bad job,” but “we know you had a tough day, but tomorrow will be better” or something.

Student 8

...just a simple update of me is good enough. Like, “good job, keep up the good work” or “good improvement.”

Student 4

#### Barriers From a Student Perspective

Three out of the 12 student participants expressed their concerns about not being able to check on their behavioral targets on a regular basis. Student 7 stated:

When I’m depressed, I don’t even want to take a look at the data or the problems, or what this could be.

Student 7

Similarly, Student 8 did not feel confident to go through the data by himself.

I don’t think I would check the tracked data all the time. It might be easier for me to go through the data with my doctor, so he can tell me what to do from there.

Student 8

**Table 1:** Summary of themes from interviews with clinician and student participants.

Themes		Subthemes
**Usefulness of the mobile sensing platform**		
	Clinician	Objective feedback to students Objective evidence to clinicians Filling communication gaps
	Student	Support continued self-tracking
**How to provide behavioral targets**		
	Clinician	Provision under clinicians’ guidance Presentation oriented by personal goals
	Student	Preference of integrated behavioral targets
**How to provide feedback**		
	Clinician	Positive reinforcement Reflection reminder Challenge negative thoughts
	Student	Avoid negative but seek positive feedback

Table 1 provides a summary of major themes extracted from interviews with clinician and student participants.

During the interview, we prompted students with questions about privacy as a potential barrier of using iSee. Nine student participants did not feel privacy was an issue as long as their data were “anonymous.” Eleven students were satisfied with the privacy issue when the researcher explained that iSee would deidentify personal data before transmitting to the cloud where the data would be stored. One student would like an option to choose what data to share:

...maybe some people want to make certain things private. Like I don’t want people knowing how much I’m sleep. It would be a good idea to allow for that so people can make it function that way.

## Discussion

### Principal Findings and Implications

This study explored the types of behavioral targets, methods of delivering feedback, and user concerns with a mobile sensing platform, iSee, from both clinician and student perspectives. Our findings show that clinicians and students recognized the benefits of the mobile sensing platform in terms of providing objective behavioral data, filling clinician-student communication gaps, and easing continued self-tracking practice. Although clinicians and students shared thoughts on potential usefulness of iSee, they differed on preferences for the types of behavioral targets and types of feedback.

#### Individual Versus Integrated Behavioral Targets

Clinician and student participants expressed different views on how to provide behavioral targets. Clinicians emphasized presenting individual behavioral targets in relation to corresponding behavioral goals, whereas students would like to see integrated behavioral targets in relation to their depression conditions. Clinicians saw value in setting up appropriate behavioral goals that are small and achievable, such as exercising 30 minutes three days a week or sleeping for 8 hours every night. They were interested in using visualizations of sensed behavioral targets to observe how much progress students have made from their baseline behaviors to the specific goals over time. In comparison, student participants wanted to understand the correlations between clusters of behaviors and their distress. For example, a student participant may be more interested in knowing how waking up during the night and lack of deep sleep are related to a depressed feeling next day than knowing that she slept only 4 hours yesterday. This requires a juxtaposition of several behavioral targets (eg, wake up, deep sleep, sleep length) and their associations with levels of depression.

The different viewpoints between clinicians and students may be due to their different objectives, which may require different design approaches. Clinicians frequently make behavioral recommendations, and much of treatment involves encouraging patients to make those changes. Thus, tools that support such monitoring may support clinicians in being more effective in their roles. According to existing literature, students are interested in self-experimentation and learn self-management skills from personal experience [19,20,21]. Therefore, for students who may not be fully aware of the associations between their behaviors and depression, it is critical to provide integrated data visualizations to facilitate sense making of behavioral targets. For students who have the awareness of how their behaviors affect depression, they might be more motivated to set up specific behavioral goals and review relevant behavioral targets for achieving the goals. While the goals of clinicians and student participants may require different design approaches, they are compatible.

#### Positive Versus Negative Feedback

Both clinicians and students embraced positive feedback when a student was making good progress. Messages that confirmed one’s achievement, validated one’s self-worth, and encouraged continued efforts were welcomed. However, clinicians and students differed to some extent in terms of whether or not providing feedback when students were not making positive progress. Students generally declined to review the behavioral targets when it did not show any positive change because the situation may make them feel depressed. This finding was consistent with existing literature on self-trackers’ experiences. For example, one study found that self-trackers experienced frustration and anxiety when they were aware of negative tracked data [20].

Clinicians presented more diverse opinions about this issue. Some clinicians suggested using gentle reminders or reflection questions to raise students’ attention to the issue reflected in negative data. Some clinicians suggested only showing negative data to students during counseling sessions so that clinicians could discuss the data with students. As suggested in the self-tracking literature, designers could build tools that customize the individual user experience [22]. Applied to the current study, the platform could solicit students’ preferences with regard to sharing and discussing negative data with their clinicians, receiving gentle reminders without presenting the actual behavioral data, or dismissing any negative information. Future research is encouraged to ask perspective-taking questions so that clinicians and students could stand in each other’s shoes and see whether a more shared perspective could emerge.

#### Concerns About Reviewing Data

While iSee attempts to reduce the burden on students of entering data, both students and clinicians nevertheless saw reviewing the data as a potential burden. Clinicians expressed concerns about not having enough time to review students’ tracked data, while students were worried that they might not be able to check on their data when they were too depressed. Some students preferred that their clinicians take the primary role of checking on their data. These concerns reflect a barrier of effectively using iSee in the clinical setting: some uncertainty in the capacity to pay attention to behavioral targets.

A couple of steps could be taken to resolve this potential barrier. For clinicians, designers are encouraged to decrease the overhead of reviewing data and using iSee. More research should be conducted to establish a stronger confidence in behavior targets that are clinically meaningful so that clinicians can make quick relevance of the data [7]. While clinicians are protective of their time, they would spare time on the platform if they believe it is beneficial to counseling [23]. In addition, sending encouragement and motivational messages may not be a good use of clinicians’ expertise [23]. Therefore, iSee could automate the process of sending supportive messages based on users’ tracked data. For students, designers might consider using push methods, such as sending data visualizations and feedback via text messages [24], instead of expecting students to open the app and review it. Moreover, tailoring data review to students’ needs (eg, what to review, how frequently to review) could give students a sense of ownership and control [23] that enhances students’ engagement with the system.

### Limitations and Future Research

Our study has several limitations. First, the study sample is small, and all participants were recruited from only one university counseling center. Caution should be taken when generalizing the interview results to clinicians and students from other university counseling centers and college students with depression. Nevertheless, the study site MSU is a large public university in the US; and, therefore, should share important characteristics with counseling centers, students, and clinicians from other public universities.

Another limitation is that our sample did not include any students with severe depression. More severely depressed students may have potentially different opinions regarding the use of iSee.

The third limitation of the study is the lack of field deployment. Given the exploratory nature of the study, we only presented the conceptual prototype of iSee to interviewees to solicit their thoughts and opinions. User experience may vary when they use the actual platform. For example, the quality of collected personal data could depend on the system design and implementation in the field deployment. The benefits of iSee and useful types of behavioral target data and feedback will have to be validated once the iSee is developed and deployed. The effectiveness of design features can only be evaluated in a randomized controlled trial of the iSee system.

This study has explored potential feedback to deliver to students with depression (eg, positive feedback, reflection reminders). The next stage of research could examine how the sensor data and behavioral targets could inform the design and delivery of micro-interventions. For example, how frequently and when should an encouraging message be sent to the user? This will require extensive user testing of different design ideas and prototypes. Future work is encouraged to connect sensor data, behavioral targets, feedback, and interventions.

Finally, we also note that this study focused only on behavioral targets where there is sufficient evidence that they can be accurately sensed using mobile phone sensors. While it is possible that these findings may extend to self-reported data, we would caution against extending this to behavioral targets not explored in this study.

### Conclusion

By conducting interviews with clinicians and student participants, we have explored the issues surrounding benefits of iSee, and useful types of behavioral target data and feedback. We have gained some initial insights such that the behavioral data generated by iSee could complement students’ activities and behaviors self-reported during counseling sessions, fill in clinician-student communication gaps, and extend therapy beyond the clinical settings by delivering appropriate feedback. With respect to preferred types of behavioral targets, we have learned that clinicians may focus on individual behavior targets with a set goal, whereas students may prefer integrated behavioral targets that assist their understanding of the relationship between their behaviors and depression. In addition, clinicians may have diverse opinions about presenting negative data to patients, whereas students try to avoid negative feedback. This qualitative work represents the first effort to understand the benefits and user needs of a mobile sensing platform such as iSee in university counseling service.

## Appendix

Multimedia Appendix 1 Interview protocols used for clinician and student participants.

## Abbreviations

GPS: global positioning system
MSU: Michigan State University

## References

[1] American College Health Association (2008). American College Health Association-National College Health Assessment Spring 2007 Reference Group Data Report (Abridged). Journal of American College Health.

[2] Buchanan JL (2012). Prevention of depression in the college student population: a review of the literature. Arch Psychiatr Nurs.

[3] Reetz D, Bershad C, LeViness P, Whitlock M (2016). www.aucccd.org.

[4] Miller-Matero LR, Clark KB, Brescacin C, Dubaybo H, Willens DE (2016). Depression and literacy are important factors for missed appointments. Psychol Health Med.

[5] Haerizadeh M, Moise N, Chang B, Edmondson D, Kronish I (2016). Depression and doctor-patient communication in the emergency department. Gen Hosp Psychiatry.

[6] Schiepek G, Aichhorn W, Gruber M, Strunk G, Bachler E, Aas B (2016). Real-Time Monitoring of Psychotherapeutic Processes: Concept and Compliance. Front Psychol.

[7] Mohr DC, Zhang M, Schueller SM (2017). Personal Sensing: Understanding Mental Health Using Ubiquitous Sensors and Machine Learning. Annu Rev Clin Psychol.

[8] Wang R, Aung M, Abdullah S, Brian R, Campbell AT, Choudhury T, Hauser M, Kane J, Merrill M, Scherer EA, Tseng V, Ben-Zeev D (2016). CrossCheck: toward passive sensing and detection of mental health changes in people with schizophrenia.

[9] Saeb S, Zhang M, Kwasny M, Karr C, Kording K, Mohr D (2015). The Relationship between Clinical, Momentary, and Sensor-based Assessment of Depression. Int Conf Pervasive Comput Technol Healthc.

[10] Saeb S, Zhang M, Karr C, Schueller S, Corden M, Kording K, Mohr D (2015). Mobile Phone Sensor Correlates of Depressive Symptom Severity in Daily-Life Behavior: An Exploratory Study. J Med Internet Res.

[11] Gravenhorst F, Muaremi A, Bardram J, Grünerbl A, Mayora O, Wurzer G, Frost M, Osmani V, Arnrich B, Lukowicz P, Tröster G (2014). Mobile phones as medical devices in mental disorder treatment: an overview. Pers Ubiquit Comput.

[12] Wang R, Chen F, Chen Z, Li T, Harari G, Tignor S, Zhou X, Ben-Zeev D, Campbell AT (2014). StudentLife: assessing mental health, academic performance and behavioral trends of college students using smartphones.

[13] Donker T, Straten AV, Marks I, Cuijpers P (2009). A Brief Web-Based Screening Questionnaire for Common Mental Disorders: Development and Validation. J Med Internet Res.

[14] Riper H, Andersson G, Christensen H, Cuijpers P, Lange A, Eysenbach G (2010). Theme issue on e-mental health: a growing field in internet research. J Med Internet Res.

[15] Wahle F, Bollhalder L, Kowatsch T, Fleisch E (2017). Toward the Design of Evidence-Based Mental Health Information Systems for People With Depression: A Systematic Literature Review and Meta-Analysis. J Med Internet Res.

[16] Kim J, Lim S, Min YH, Shin Y, Lee B, Sohn G, Jung KH, Lee J, Son BH, Ahn SH, Shin S, Lee JW (2016). Depression Screening Using Daily Mental-Health Ratings from a Smartphone Application for Breast Cancer Patients. J Med Internet Res.

[17] Braun V, Clarke V (2006). Using thematic analysis in psychology. Qualitative Research in Psychology.

[18] Cohen S, Hoberman HM (1983). Positive Events and Social Supports as Buffers of Life Change Stress1. J Appl Social Pyschol.

[19] Karkar R, Zia J, Vilardaga R, Mishra SR, Fogarty J, Munson SA, Kientz JA (2016). A framework for self-experimentation in personalized health. J Am Med Inform Assoc.

[20] Choe EK, Lee Nicole, Lee Bongshin, Pratt Wanda, Kientz JA (2014). Understanding quantified-selfers' practices in collecting and exploring personal data.

[21] Ravichandran R, Sien S, Patel SN, Kientz JA, Pina LR (2017). Making sense of sleep sensors: How sleep sensing technologies support and undermine sleep health.

[22] Arean PA, Hallgren KA, Jordan JT, Gazzaley A, Atkins DC, Heagerty PJ, Anguera JA (2016). The Use and Effectiveness of Mobile Apps for Depression: Results From a Fully Remote Clinical Trial. J Med Internet Res.

[23] Doherty Gavin, Coyle David, Sharry John (2012). Engagement with online mental health interventions: an exploratory clinical study of a treatment for depression.

[24] Bardram Jakob E., Frost Mads, Szanto Karoly, Vinberg Maj, Kessing Lars Vedel (2013). Designing mobile health technology for bipolar disorder: a field trial of the monarca system.

