# Supplementary material for: Exploring User Needs for a Mobile Behavioral-Sensing Technology for Depression Management: Qualitative Study
Source: J Med Internet Res. 2018 Jul 17;20(7):e10139. doi: 10.2196/10139 (PMC6068382; doi:10.2196/10139)
Supplement: Multimedia Appendix 1 [file jmir_v20i7e10139_app1.pdf]

Interview Protocol for Clinician Participants

Welcome

I'm [interviewer name]. As you sit down, please take some time to read through the consent form in front of you before you sign it. Please let me know if you have any questions or if you would like me to read through the consent form with you. We'll go through the main points after finishing reading the consent form.

Background

- Thank you for taking the time to meet with us.
- I'm going to audio record the interview so that I can go back later and listen to it. When our research team publishes papers using this data, you will not be named or identified in any way.
- As you read in the consent form, the purpose of this interview is to learn about what clinicians think about using a mobile behavioral sensing platform in their counseling students with depression. Please feel free to express your true thoughts and opinions. There are no right or wrong answers.
- At the end of the interview, you will get $20 cash for your participation.

Introduction

- Communication College and Engineering College are collaborating to develop a mobile behavioral sensing platform. This platform includes sensing and analytics technology, and a web portal for clinicians to view and interact with outputs.
- This platform uses sensors on smartphones and wristbands to unobtrusively track student clients' physical activity, travel, phone use, eating behavior and sleep behavior (Show Figure 1). Then, algorithms will run and generate data outputs related to those behaviors that I just mentioned. We hope to deliver these data to you (Show Figure 2). This figure is only an example of data outputs. In this interview, we want to learn what kind of data and outputs would be helpful to you.

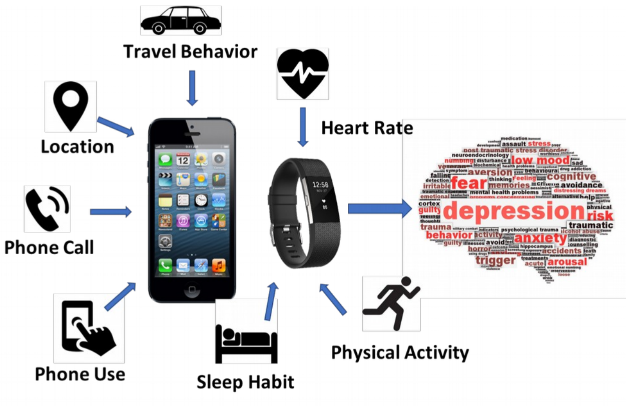

Figure1 Behaviors that the mobile sensing platform will track

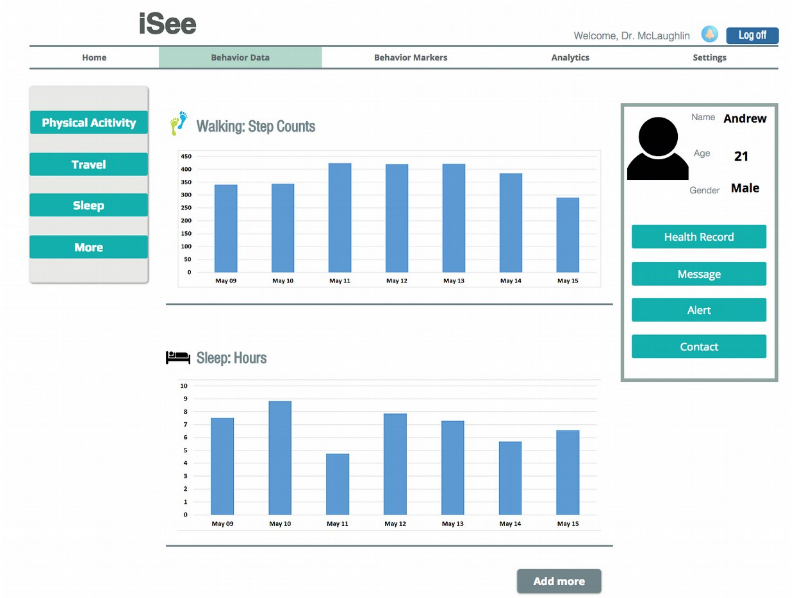

Figure 2 An example of outputs on the web portal for clinicians

Interview Question Begins:

- So let's begin the interview. First, we want to know your thoughts and opinions about how this mobile sensing platform could be useful or beneficial to counseling services provided to students with depression.
  - Prompts:
    - Are there certain challenges in your counseling practice that having this mobile sensing platform would help?
    - Have you asked your student clients to track or keep a log on their behavior?

- What behavioral data do you think most clinically useful? That is, the data will be helpful for you to assess a client's depression condition and make a decision on therapy.
  - Prompts:
    - Physical activity: frequency of exercise, intensity of exercise, time spent on physical activity, sedentary activity?
    - Eating behavior: frequency of eating, timing of eating, regularity?
    - Sleep behavior: amount and quality of sleep?
    - Travel: physical locations?
    - Phone use: time spent on cell phone?
    - Social interactions: frequency and time spent with interacting with others, the nature/type of interaction (e.g., pleasant or negative)?
    - In what format? How to visualize the data?

- How much or what type of behavioral data do you think will be helpful to present to student clients?

- Prompts:
  - Why is it helpful?
  - Timing and frequency of delivery?
  - Positive or negative feedback?
  - In what format of presentation?

- What barriers do you expect to use this platform at your work?
  - Prompts:
    - How can this platform be integrated into your work routines?
    - What concerns do you have for student clients to use this platform?
    - What concerns do you have for clinicians to use this platform?

==

Interview Protocol for Student Participants

Welcome

I'm [interviewer name]. As you sit down, please take some time to read through the consent form in front of you before you sign it. Please let me know if you have any questions or if you would like me to read through the consent form with you. We'll go through the main points after finishing reading the consent form.

Background

- Thank you for taking the time to meet with us.
- I'm going to audio record the interview so that I can go back later and listen to it. When our research team publishes papers using this data, you will not be named or identified in any way.
- As you read in the consent form, <u>the purpose of this interview is to learn about your opinion about using a mobile behavioral sensing platform to manage your depression condition.</u> Please feel free to express your true thoughts and opinions. There are no right or wrong answers.
- At the end of the interview, you will get $20 cash for your participation.

Introduction

- Communication College and Engineering College are collaborating to develop a mobile behavioral sensing platform. This platform includes sensing and analytics technology, and mobile app for students to view and interact with outputs.
- This platform uses sensors on smartphones and wristbands to unobtrusively track your physical activity, travel, phone use, eating behavior and sleep behavior (Show Figure 1). So you do not need to manually input the data. Then, algorithms will run and generate data outputs related to those behaviors that I just mentioned.

We hope to deliver these data to you (Show Figure 2). This screen shot is only an example of data outputs. In this interview, we want to learn what kind of data and outputs would be helpful to you.

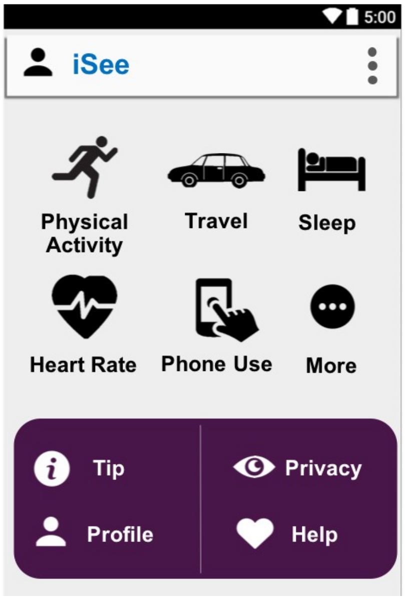

Figure 1 Behaviors that the mobile sensing platform will track

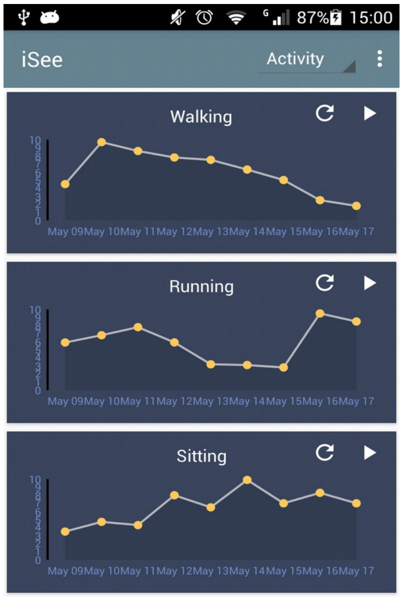

Figure 2 An example of outputs on the mobile app for students

Interview Question Begins:

- So let's begin the interview. First, we want to know your thoughts and opinions about how this mobile sensing platform could be helpful to manage your depression condition.
  - Prompts:
    - When you cope with depression, what do you find the most difficult to deal with?
    - Have you ever tracked your behavior to manage your depression?

- What behavioral data and feedback do you think helpful to manage your depression condition? and how?
  - Prompts:
    - Physical activity: frequency of exercise, intensity of exercise, time spent on physical activity, sedentary activity?
    - Eating behavior: frequency of eating, timing of eating, regularity?
    - Sleep behavior: amount and quality of sleep?
    - Travel: physical locations?
    - Phone use: time spent on cell phone?

        - Social interactions: frequency and time spent with interacting with others, the nature/type of interaction (e.g., pleasant or negative)?

- How do you want behavioral data and feedback to be presented to you?
    - Prompts:
        - In what format?
        - Positive or negative feedback?
        - How frequently and how likely are you to read?

- What barriers do you expect to use this platform in your daily life?
    - Prompts:
        - Are you concerned about privacy?
        - Do you feel comfortable to share data with clinicians? If not, what kind of information you want to withhold?
